# Blockade of insulin-like growth factors increases efficacy of paclitaxel in metastatic breast cancer

**DOI:** 10.1038/s41388-017-0115-x

**Published:** 2018-01-25

**Authors:** Lucy Ireland, Almudena Santos, Fiona Campbell, Carlos Figueiredo, Dean Hammond, Lesley G. Ellies, Ulrike Weyer-Czernilofsky, Thomas Bogenrieder, Michael Schmid, Ainhoa Mielgo

**Affiliations:** 1 Department of Molecular and Clinical Cancer Medicine, University of Liverpool, Liverpool, UK; 2 Department of Physiology, University of Liverpool, Liverpool, UK; 3 Department of Pathology, University of California San Diego, La Jolla, USA; 4 Boehringer Ingelheim RCV GmbH & Co KG, Pharmacology and Translational Research, Vienna, Austria; 5 Boehringer Ingelheim RCV GmbH & Co KG Medicine and Translational Research, Vienna, Austria; 6 Department of Urology, University Hospital Grosshadern, Ludwig-Maximilians-University, Munich, Germany

## Abstract

Breast cancer remains the leading cause of cancer death in women owing to metastasis and the development of resistance to established therapies. Macrophages are the most abundant immune cells in the breast tumor microenvironment and can both inhibit and support cancer progression. Thus, gaining a better understanding of how macrophages support cancer could lead to the development of more effective therapies. In this study, we find that breast cancer-associated macrophages express high levels of insulin-like growth factors 1 and 2 (IGFs) and are the main source of IGFs within both primary and metastatic tumors. In total, 75% of breast cancer patients show activation of insulin/IGF-1 receptor signaling and this correlates with increased macrophage infiltration and advanced tumor stage. In patients with invasive breast cancer, activation of Insulin/IGF-1 receptors increased to 87%. Blocking IGF in combination with paclitaxel, a chemotherapeutic agent commonly used to treat breast cancer, showed a significant reduction in tumor cell proliferation and lung metastasis in pre-clinical breast cancer models compared to paclitaxel monotherapy. Our findings provide the rationale for further developing the combination of paclitaxel with IGF blockers for the treatment of invasive breast cancer, and Insulin/IGF1R activation and IGF+ stroma cells as potential biomarker candidates for further evaluation.

## Introduction

Breast cancer is the leading cause of cancer death in females worldwide, and is characterized by a high proliferation rate, an increased capacity to metastasize, and its ability to resist standard therapies [1]. Triple-negative breast cancer (TNBC) is a highly metastatic subtype of breast cancer that accounts for ~ 20% of all breast cancer cases and has limited efficacious treatment options [2]. Current standard treatments for metastatic disease include radiotherapy and chemotherapy [3, 4]. TNBC has a poorer survival rate, its biology is comparatively less well-understood and currently no effective specific targeted therapy is readily available [5]. Breast cancer has a propensity to give rise to distant metastasis at sites such as the lungs, bone, and brain, which can present up to 10 years after treatment [6]. Patients with metastatic breast cancer ultimately often become resistant to current chemotherapy treatments and as a result account for >90% of breast cancer deaths [7], highlighting the need for new therapeutic targets to treat metastatic burden more effectively.

Tumor progression and response to therapy is not only dependent on tumor intrinsic mechanisms but also involves modulation by surrounding non-malignant stromal cells in the tumor microenvironment [8, 9]. Macrophages are the most abundant leukocytes in the breast tumor microenvironment [10] and an increase in tumor-associated macrophages (TAMs) correlates with a poorer prognosis in patients [11–13]. Macrophages can be polarized into M1-like anti-tumorigenic macrophages and M2-like pro-tumorigenic macrophages [14–16]. M2-like macrophages can influence tumor initiation, progression, metastasis [17–19], and resistance to therapies [20–22].

Cancer progression relies on the continued propagation of cancer cells, which can be stimulated by external ligands activating signaling pathways of tumor cell survival and proliferation, even when challenged with chemotherapy [23–26]. The insulin-like growth factor (IGF) signaling axis has been implicated in promoting cancer progression in several tumor types including breast cancer [27–29], and in breast cancer resistance to estrogen and HER2 receptor inhibition [27, 30–32]. Interestingly, Fagan et al. [33] showed that tamoxifen-resistant ER+ cells showed a reduction in the number of IGF-1 receptors, whereas the number of insulin receptors and AKT phosphorylation levels remained unaltered when stimulated with Insulin and IGF-2, suggesting that both IGF-1 and IGF-2 signaling may support resistance of breast cancer cells to therapies. However, the role of IGF signaling in tumor progression and resistance to chemotherapy in breast cancer is not completely understood yet [32]. We and others have recently shown that stroma-derived IGFs promote survival of cancer cells leading to therapy resistance in pancreatic and brain cancer models, respectively [22, 34]. In the current studies, we aimed to investigate the role of stroma-derived IGF in breast cancer progression and metastasis, and to explore the therapeutic opportunity of blocking IGF signaling in combination with chemotherapy for the treatment of breast cancer.

## Results

### Insulin and IGF-1 receptors are activated on tumor cells in biopsies from breast cancer patients, and this positively correlates with increased TAM infiltration and advanced tumor stage

**Fig. 1:**
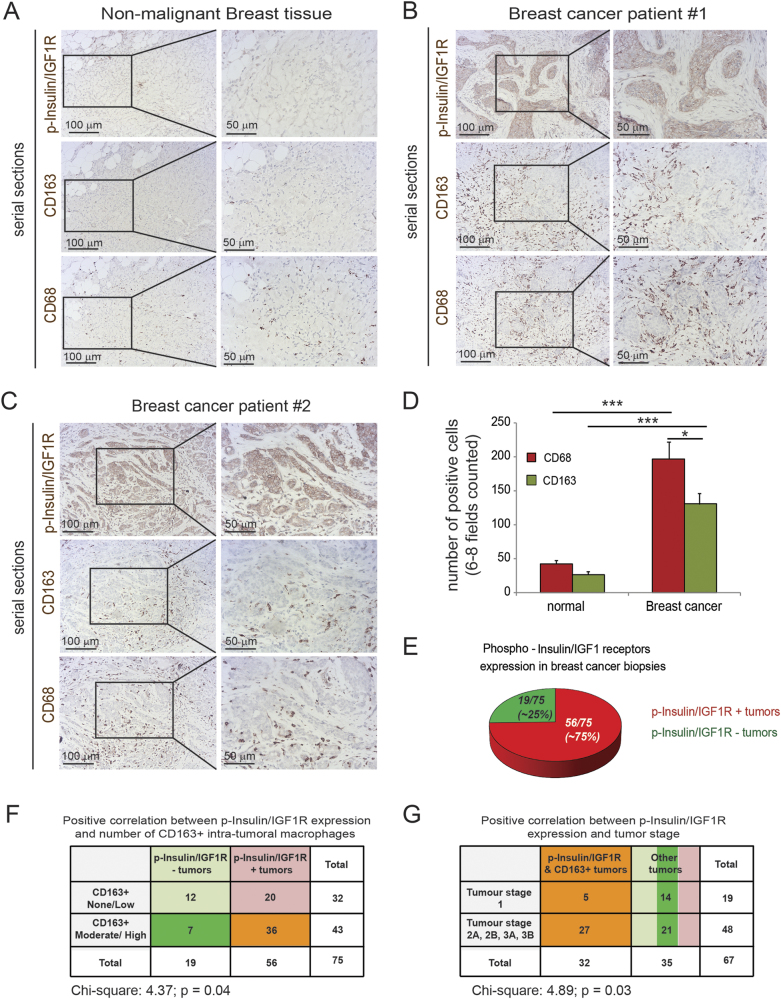
75% of breast cancer patients have activated Insulin/IGF1 receptors and Insulin/IGF-1 receptor activation positively correlates with macrophage infiltration and advanced tumor stage. **a** Serial sections of biopsies from non-malignant breast tissue immunohistochemically stained for phospho-Insulin/IGF1 receptor, CD163 and CD68. Scale bars, 100 μm and 50 μm. **b** and **c** Serial sections of biopsies from breast cancer patients immunohistochemically stained for phospho-insulin/IGF1 receptor, CD163, and CD68. Scale bars, 100 μm and 50 μm. **d** Bar graph depicting the quantification of CD68 and CD163 positive macrophages in non-malignant breast tissue and breast cancer tissue samples. Error bars represent s.d. (*n* = 3); * two-tailed *p*-value ≤ 0.05, *** two-tailed *p*-value ≤ 0.005 using a student’s *t*-test. **e** Pie diagram representing the percentage of phospho-Insulin/IGF-1 receptor positive (red) and negative (green) tumors assessed in a tissue microarray containing biopsies from 75 breast cancer patients. **f** Contingency table and results from statistical analysis showing a positive correlation between phospho-Insulin/IGF-1 receptor expression in breast tumors and increased CD163+ macrophage infiltration. Chi-square = 4.37; *p* = 0.04. **g** Contingency table and results from statistical analysis showing a positive correlation between phospho-Insulin/IGF1 receptor and CD163+ macrophages co-expression and tumor stage. Chi-square = 4.89; *p* = 0.03

Macrophages have an important role in breast cancer progression and metastasis [35, 36] and have been shown to express high levels of IGFs in other cancer types [22, 34], but the role of IGF-expressing macrophages in breast cancer has not yet been explored. To investigate whether IGF-signaling pathways are activated in invasive breast cancer progression and whether their activation correlates with macrophage infiltration, we first evaluated the activation status of insulin and IGF-1 receptors in biopsies from breast cancer patients, and the levels of infiltrated TAMs. Immunohistochemical staining of serial sections of non-malignant breast tissue (Fig. 1a) and breast cancer patients’ tissues (Fig. 1b, c) revealed an increase in phospho-insulin/IGF-1 receptor levels in the breast cancer tissues along with increased infiltration of CD68+ (pan-myeloid/macrophage marker) and CD163+ (marker commonly used to identify pro-tumorigenic M2-like macrophages) macrophages (Fig. 1a–d). Analysis of a tissue microarray (TMA) containing samples from 75 breast cancer patients, with different tumor stages but unspecified subtype, showed that Insulin/IGF-1R signaling was activated in 56 of 75 (~ 75%) patients (Fig. 1e and Supplementary Table. S1). Activation of insulin/IGF-1 receptors positively correlates with increased infiltration of CD163+ macrophages in the tumor (Fig. 1f) and with advanced tumor stage (Fig.1g).

### 87% of patients with invasive breast cancer have insulin/IGF-1 receptors activated

**Fig. 2:**
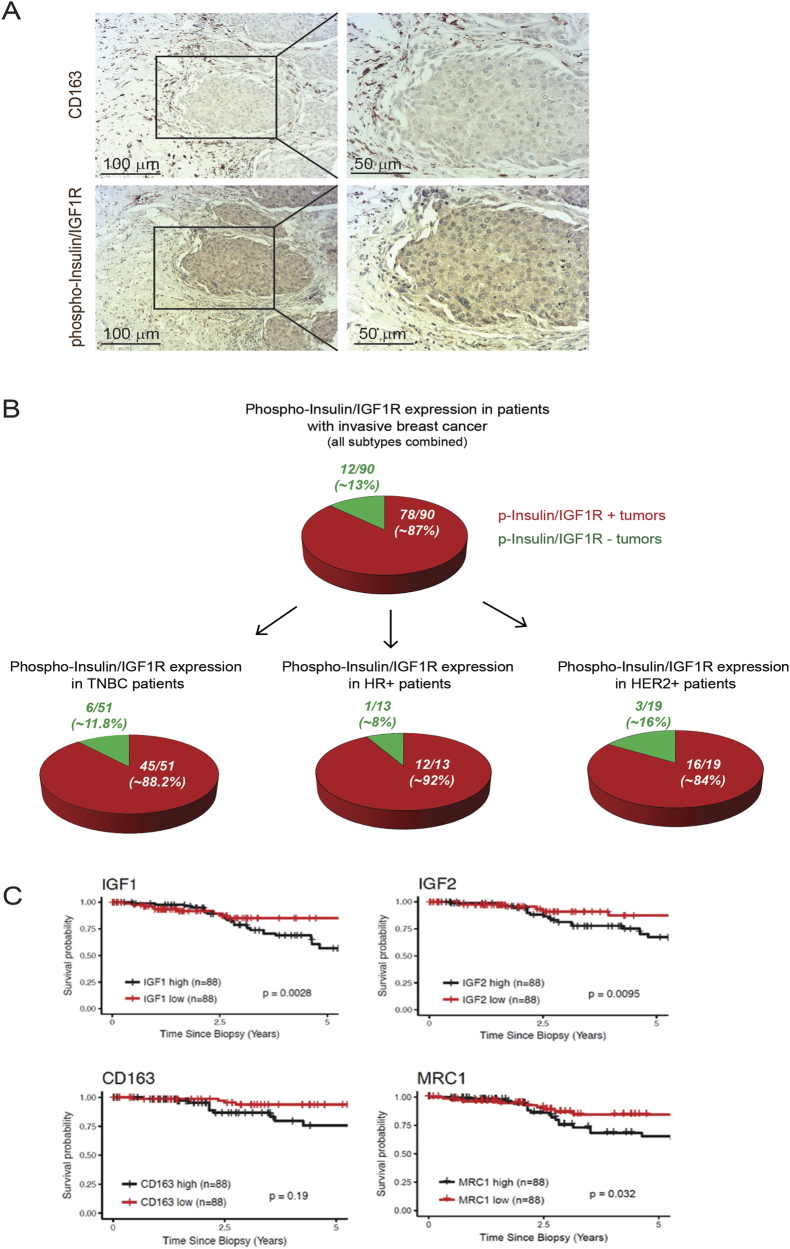
Eighty-seven percent of patients with invasive breast cancer have activated Insulin/IGF1 receptors. **a** Immunohistochemical staining of invasive breast cancer tissue serial sections stained for phospho-Insulin/IGF1 receptor and CD163. Scale bars, 100 μm and 50 μm. **b** Upper diagram: Pie diagram representing the percentage of phospho-Insulin/IGF1 receptor positive (red) and negative (green) tumors assessed in tissue microarray (TMA) containing biopsies from 90 consented patients with invasive breast cancer. Lower diagrams: represent the percentage of phospho-Insulin/IGF1 receptor positive (red) and negative (green) tumors of the molecular subsets, TNBC, HR+, and HER2+. **c** Expression levels of *Igf-1*, *Igf-2*, *cd163*, and *mrc1* associated with survival in breast cancer patients

As insulin/IGF-1 receptors activation positively correlates with advanced tumor stage, we further analyzed biopsies from patients with invasive breast cancer. Immunohistochemical staining revealed that invasive breast cancer also shows increased phospho-insulin/IGF-1 receptor levels in tumor cells surrounded by CD163+ macrophages (Fig. 2a). Analysis of a TMA containing 90 samples from patients with invasive breast cancer showed that 78 of 90 (~ 87%) of these patients have Insulin/IGF1 receptors activated (Fig. 2b, upper pie diagram, and Supplementary Table S2). Among these 90 samples, 51 were TNBC of which 45 (~ 88.2%) showed activation of Insulin/IGF1 receptors (Fig. 2b, lower, left pie diagram), 13 were hormone-receptor positive (HR+) of which 12 (~ 92%) showed activation of Insulin/IGF1 receptors (Fig. 2b, lower, middle pie diagram), and 19 were HER2 positive (HER2+) of which 16 (~ 84%) showed activation of Insulin/IGF1 receptors (Fig. 2b, lower, right pie diagram). Using the cancer genome atlas database we also found that increased gene expression of *Igf-1*, *Igf-2*, and the M2-like macrophage markers *cd163* and *mrc1* positively correlates with reduced survival in breast cancer patients (Fig. 2c). Together these results suggest an important role for IGF signaling in invasive breast cancer of all subtypes, including TNBC.

### TAMs and fibroblasts are the main sources of IGF-1 and IGF-2 in invasive breast cancer

**Fig. 3:**
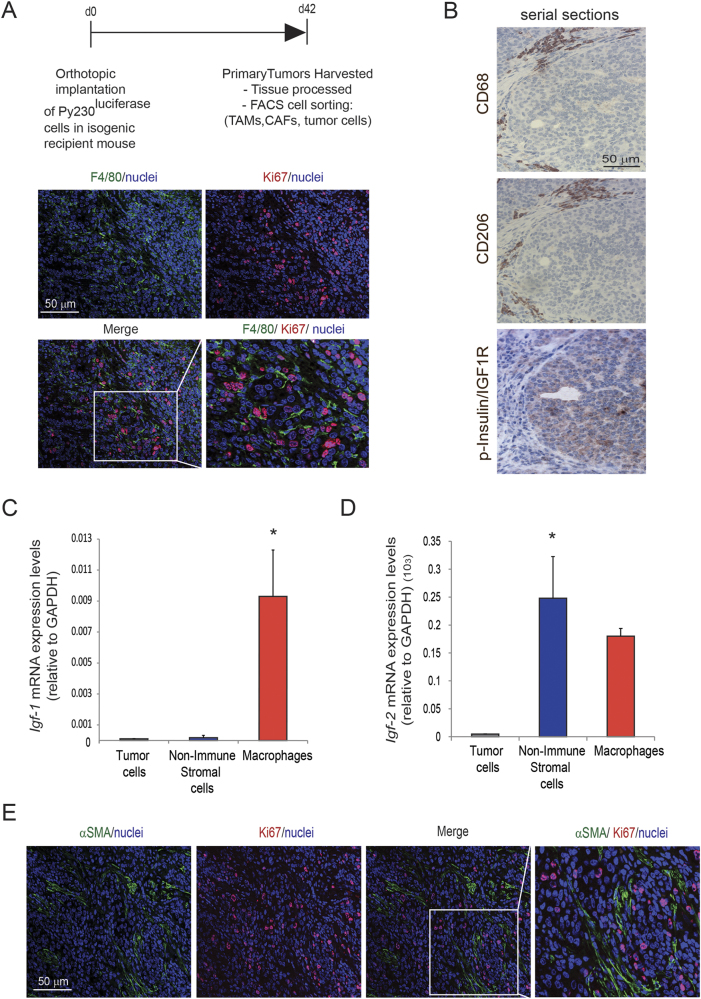
TAMs and CAFs are the main sources of IGF-1 and IGF-2 in invasive breast adenocarcinomas. **a** PY230 tumor cells were subcutaneously implanted into the third mammary gland of syngeneic recipient mice. Images show immunofluorescent staining for F4/80 (green), Ki67 (red), and nuclei (blue) in murine breast cancer tissue harvested at day 42 after tumor implantation. Scale bar 50 μm. **b** Serial sections of immunohistochemical staining for CD68, CD206 and phospho-Insulin/IGF1 receptor in murine breast tumors. Scale bar 50 μm. **c** *Igf-1* mRNA expression levels were quantified in tumor cells, non-immune stromal cells and tumor-associated macrophages isolated from murine breast cancer tumors by flow cytometry. Error bars represent s.e. (n = 3), * *p*-value ≤ 0.05 using one-way ANOVA and Bonferroni post hoc test. **d** *Igf-2* mRNA expression levels were quantified in tumor cells, non-immune stromal cells and tumor-associated macrophages isolated from murine breast cancer tumors by flow cytometry. Error bars represent s.e. (*n* = 3), * *p*-value ≤ 0.05 using one-way ANOVA and Bonferroni post hoc test. **e** Immunofluorescent images of αSMA (green), Ki67 (red), and nuclei (blue) in breast tumors. Scale bar 50 μm

To further understand the correlation between activation of Insulin/IGF1 receptors and increased TAMs infiltration, we used an orthotopic syngeneic TNBC pre-clinical model, which has been shown to recapitulate the human disease progression [37]. In brief, we isolated murine breast cancer cells (Py230) from the genetically engineered spontaneous breast cancer model MMTV-PyMT and transduced isolated cells with a reporter lentivirus expressing zsGreen/luciferase allowing in vivo imaging of tumor burden. To induce breast tumor burden, Py230zsGreen/luciferase cells were orthotopically implanted into the mammary fatpad of isogenic immunocompetent recipient mice. Forty-two days after implantation, primary tumors were surgically removed and tumor tissue sections were analyzed by immunofluorescent and immunohistochemical staining. We found that tumors were highly infiltrated by macrophages (F4/80+, CD68+, CD206+) that surround proliferating tumor cells (Ki67+) (Fig. 3a), which also express phosphorylated/activated insulin/IGF-1 receptors (Fig. 3b). Thus, similar to what we observed in human biopsies, murine breast cancer cells, which are spatially located in close proximity to M2-like TAMs, have activated Insulin/IGF-1 receptors.

Next, we aimed to identify the source of insulin and IGF-1 receptors ligands, namely IGF-1 and IGF-2, in the tumor microenvironment. Therefore, we enzymatically digested primary tumors to prepare single cells suspensions and isolated tumor cells, non-immune stromal cells and macrophages, by flow cytometry cell sorting (Supplementary Fig. S1A B). Gene expression analysis of isolated cell populations revealed that TAMs are the main source of *Igf-1* and that both TAMs and cancer-associated fibroblasts (CAFs) are major sources of *Igf*-2 in the breast tumor microenvironment (Fig. 3c, d). Immunofluorescent staining of αSMA and Ki67 in the murine breast tumors showed that αSMA+ stromal cells, which appear to be a major source of IGF-2, also surround actively dividing tumor cells (Fig. 3e). IGF-1 and IGF-2 are also expressed in the stroma surrounding cancer cells in human TNBC samples (Supplementary Fig. S2A), and gene expression analysis of *Igf-1* and *Igf-2* in human MDA-MB-231 TNBC cells and primary human macrophages revealed that human macrophages express high levels of *Igf-1* and *Igf-2*, whereas there was very little expression of these ligands in the breast cancer cells (Supplementary Fig. S2B and C).

### Metastasis-associated macrophages and fibroblasts remain the main sources of IGF-1 and IGF-2 in pulmonary metastatic lesions

**Fig. 4:**
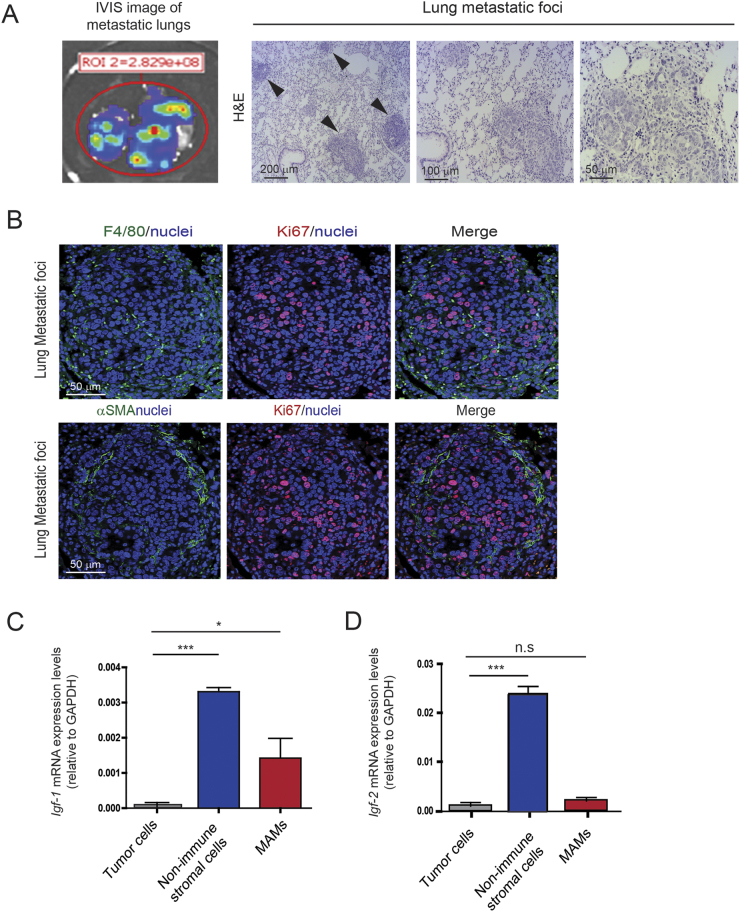
Metastasis-associated macrophages and fibroblasts express IGF-1 and IGF-2 in metastatic lungs. **a** Left, identification of metastatic tumor lesions in the lung by bioluminescent imaging technique of orthotopically implanted PY230^luc^ breast cancer cells. Right, images show H&E staining of metastatic foci in the lungs. Arrows indicate metastatic foci, scale bars 200 μm, 100μm, and 50 μm. **b** Immunofluorescent images of lung metastatic foci stained for F4/80 (green), αSMA (green), Ki67 (red), and nuclei (blue). Scale bar 50 μm. **c** Quantification of *Igf-1* mRNA expression levels in metastatic tumor cells, metastasis-associated non-immune stromal cells and metastasis-associated macrophages isolated from pulmonary metastasis. Error bars represent s.e. (*n* = 3), * *p*-value ≤ 0.05, *** *p*-value ≤ 0.0001 using one-way ANOVA and Bonferroni post hoc test. **d)** Quantification of *Igf-2* mRNA expression levels in metastatic tumor cells, metastasis-associated non-immune stromal cells and metastasis-associated macrophages isolated from pulmonary metastasis. Error bars represent s.e. (*n *= 3), *** *p-*value ≤ 0.05 using one-way ANOVA and Bonferroni post hoc test

Breast cancer is a highly invasive disease and often metastasizes to the lung. Orthotopically implanted Py230 breast cancer cells into syngeneic recipient mice effectively metastasized to the lung, where they formed metastatic tumors (Fig. 4a). We wondered whether IGF-1 and IGF-2 might also be expressed in the metastatic tumor microenvironment and thereby provide a survival/proliferative signal to disseminated cancer cells. To address this question, we analyzed metastatic lung tumors from our animal model for the expression of *Igf-1* and *Igf-2* in the metastasis-associated stromal cells.

Metastatic tumors in lungs were first confirmed by bioluminescence ex vivo imaging and by hematoxylin and eosin (H&E) staining (Fig. 4a). Immunofluorescent staining showed that pulmonary metastatic lesions are surrounded by macrophages (F4/80+) and myofibroblasts (αSMA+) (Fig. 4b). Similar to what we observe at the primary site, metastasis-associated macrophages and fibroblasts express high levels of *Igf-1* and *Igf-2*, whereas disseminated breast cancer cells do not express these ligands (Fig. 4c, d). Together, these findings provide evidence that macrophages and fibroblasts are the main sources of IGF-1 and IGF-2 both at the primary and the metastatic site in invasive breast cancer.

### Combination treatment of invasive breast cancer with paclitaxel and IGF blocking antibody reduces tumor cell proliferation and metastasis in a syngeneic orthotopic Py230 model

**Fig. 5:**
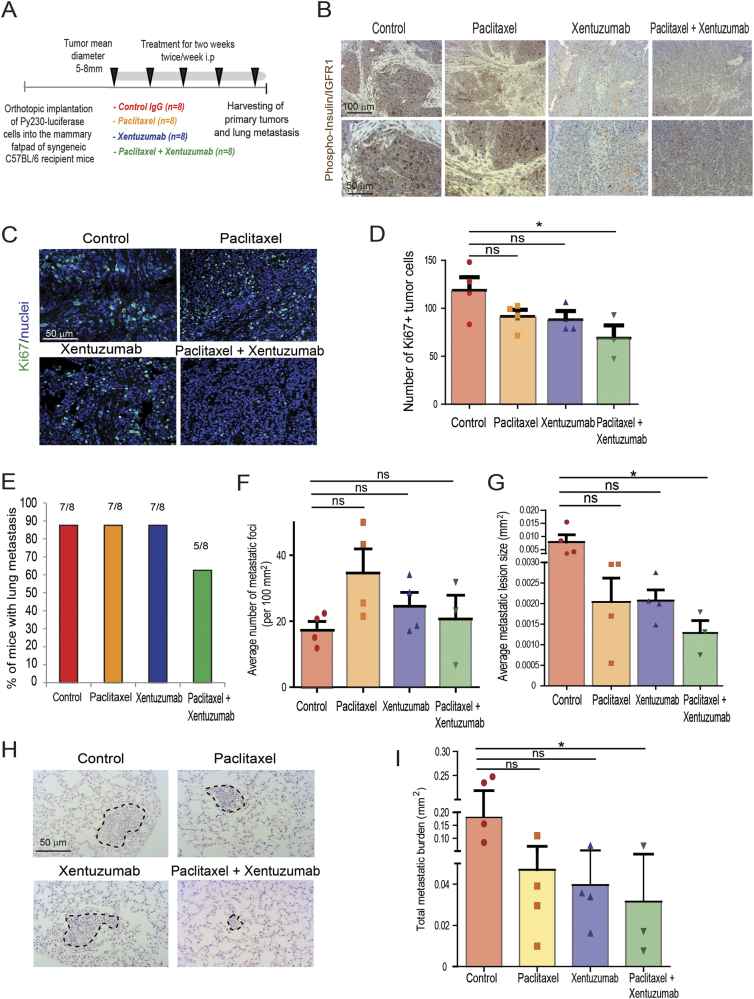
Combined treatment of IGF blocking antibody with paclitaxel decreases breast cancer proliferation and metastasis in Py230 model. **a** Py230 luciferase cells were orthotopically implanted into the third mammary fatpad of syngeneic C57BL/6 recipient mice and mice were treated, starting when tumors reached between 5–8 mm^2^, twice a week i.p., with control IgG antibody, IGF blocking antibody xentuzumab (100 mg/kg), paclitaxel (100 mg/kg), or a combination of xentuzumab with paclitaxel (*n* = 8 mice per group). **b** Immunohistochemical staining of phospho-insulin/IGF-1R in breast tumors treated with IgG (control), paclitaxel, xentuzumab or paclitaxel + xentuzumab. Scale bars 100 μm and 50 μm**. c** Immunofluorescent staining of Ki67 in primary tumors treated with IgG (control), paclitaxel, xentuzumab, or paclitaxel + xentuzumab. Scale bar 50 μm. **d** Quantification of Ki67-positive tumor cells in tumors treated with IgG (control), paclitaxel, xentuzumab or paclitaxel + xentuzumab. 3–5 fields counted/mouse tumor, *n* = 3–4 mice per treatment group, * *p* ≤ 0.05 using one-way ANOVA and Bonferroni post hoc test. **e** Percentage of mice presenting with lung metastasis per treatment group. (*n* = 8 mice/group). **f** Quantification of number of lung metastatic foci per 100mm^2^ in mice treated with control IgG, paclitaxel, xentuzumab, or paclitaxel + xentuzumab. ns, non-significant differences using one-way ANOVA and Bonferroni post hoc test. **g** Average size of pulmonary metastatic lesions (mm^2^) in mice treated with control IgG, paclitaxel, xentuzumab, or paclitaxel + xentuzumab, * *p* ≤ 0.05, using one-way ANOVA and Bonferroni post hoc test. **h** H&E staining of lung metastatic foci in mice treated with control IgG, paclitaxel, xentuzumab, or paclitaxel + xentuzumab. Scale bar 50 μm. **i** Total metastatic burden (mm^2^) in mice treated with control IgG, paclitaxel, xentuzumab, and paclitaxel with xentuzumab, * *p* ≤ 0.05, using one-way ANOVA and Bonferroni post hoc test

To determine whether IGF signaling affects breast cancer progression, metastasis and response to paclitaxel, a standard chemotherapeutic agent used to treat breast cancer, we treated mice orthotopically implanted with Py230 TNBC cells, with control IgG antibody, IGF-1/2 blocking antibody xentuzumab, paclitaxel, or xentuzumab with paclitaxel (Fig. 5a). As expected, control and paclitaxel treated mice showed high levels of insulin and IGF-1 receptor activation in the primary breast cancer tumors, whereas the xentuzumab and xentuzumab with paclitaxel treated groups showed markedly reduced levels of insulin and IGF-1 receptor activation, confirming that xentuzumab has reached the tumor and has blocked IGF signaling (Fig. 5b) [38]. No differences were seen in primary tumor growth (Supplementary Fig. S3A), in tumor cell death (Supplementary Fig. S3B) or in TAM infiltration of primary tumors (Supplementary Fig. S3C) between the different treatment groups. However, control IgG-treated mice showed higher levels of Ki67+ proliferating tumor cells, which were modestly reduced by both paclitaxel and xentuzumab single treatments and significantly reduced by the combination treatment of xentuzumab with paclitaxel (Fig. 5c, d). In addition, mice treated with the combination of xentuzumab with paclitaxel treatment showed a reduction in lung metastasis incidence (Fig. 5e). Although there were no significant differences in the number of metastatic foci in the different treatment groups (Fig. 5f), we found a significant reduction in the size of metastatic lesions and overall lung metastatic burden in the group treated with both paclitaxel and xentuzumab (Fig. 5g–i). These data suggest that initial metastatic seeding is not affected by the combination treatment, but that paclitaxel/xentuzumab treatment impairs metastatic outgrowth of disseminated breast cancer cells.

### Combination treatment of invasive breast cancer with paclitaxel and IGF blocking antibody reduces tumor cell proliferation and metastasis in a syngeneic orthotopic 4T1 model

**Fig. 6:**
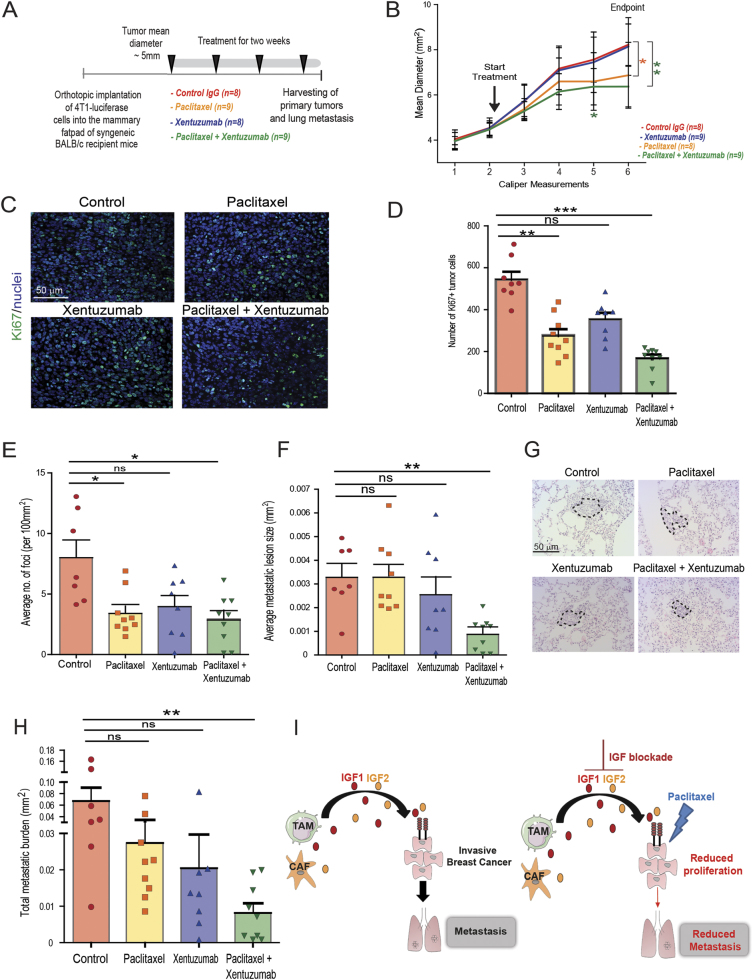
Combined IGF blocking antibody with paclitaxel decreases metastatic burden in 4T1 breast cancer model. **a** 4T1-zsgreen/luciferase cells were orthotopically implanted into the third mammary fatpad of syngeneic Balb/c recipient mice and were treated when tumors reached ~ 5 mm mean diameter, over 2 weeks the mice received four treatments by i.p. with control human IgG antibody (*n* = 8 mice), IGF blocking antibody xentuzumab (100 mg/kg) (*n* = 8 mice), paclitaxel (100 mg/kg) (*n* = 9 mice) or a combination of xentuzumab with paclitaxel (*n* = 9 mice). **b** Graph showing tumor mean diameter (mm^2^) measured by calipers before and during treatment with isotype control, xentuzumab, paclitaxel, and paclitaxel with xentuzumab. Error bars represent s.e. (IgG antibody and xentuzumab *n* = 8, paclitaxel and paclitaxel with xentuzumab *n* = 9), * *p*-value ≤ 0.05, ** *p*-value ≤ 0.01 using two-way ANOVA and Bonferroni post hoc test. **c** Immunofluorescent staining of Ki67 in primary tumors treated with IgG (control), paclitaxel, xentuzumab, or paclitaxel + xentuzumab. Scale bar 50 μm. **d** Quantification of Ki67-positive tumor cells in tumors treated with human IgG (control), paclitaxel, xentuzumab, or paclitaxel + xentuzumab. A total of 3–5 fields counted/mouse tumor, *n* = 8–9 mice per treatment group, ns, non-significant differences, ** *p* ≤ 0.01, *** ≤ 0.0001 *p* using one-way ANOVA and Bonferroni post hoc test. Images quantified using NIS-Elements Advanced Research software. **e** Quantification of number of lung metastatic foci per 100mm^2^ in mice treated with control IgG, paclitaxel, xentuzumab, or paclitaxel + xentuzumab. Error bars represent s.e. (IgG antibody *n *= 7, paclitaxel *n* = 9, xentuzumab *n* = 8, and paclitaxel with xentuzumab *n* = 9) ns, non-significant differences, * *p*-value ≤ 0.05, using one-way ANOVA and Bonferroni post hoc test. **f** Average size of pulmonary metastatic lesions (mm^2^) in mice treated with control IgG, paclitaxel, xentuzumab, or paclitaxel + xentuzumab. Error bars represent s.e., ns, non-significant differences, ** *p* ≤ 0.01, using one-way ANOVA and Bonferroni post hoc test. **g** H&E staining of lung metastatic foci in mice treated with control IgG, paclitaxel, xentuzumab, or paclitaxel + xentuzumab. Scale bar 50 μm. **h** Total metastatic burden (mm^2^) in mice treated with control IgG, paclitaxel, xentuzumab, and paclitaxel with xentuzumab. Error bars represent s.e., ns, non-significant differences, ** *p* ≤ 0.01, using one-way ANOVA and Bonferroni post hoc test. **i** Schematics describing the role of stroma-derived IGF-1 and 2 in regulating the response of metastatic breast cancer to paclitaxel

To confirm the results from the Py230 experiment in another model, 4T1-zsGreen/luciferase cells were implanted into the mammary fatpad of syngeneic Balb/c mice and treated with isotype control antibody, paclitaxel, xentuzumab, or paclitaxel with xentuzumab (Fig. 6a). In accordance with our previous findings, insulin and IGF-1R receptor activation was markedly reduced in the mice treated with xentuzumab alone and paclitaxel with xentuzumab (Supplementary Fig. S4A) [38]. In this model, we found that primary tumor growth was significantly reduced by paclitaxel treatment and even further reduced by the combination treatment paclitaxel + xentuzumab (Fig. 6b). Analysis of Ki67 + proliferating cells in the primary tumor showed a significant reduction in paclitaxel treated mice and reduced further in paclitaxel with xentuzumab treated mice compared with control IgG mice (Fig. 6c, d). The numbers of lung metastatic foci were significantly reduced by both paclitaxel alone and the combination of paclitaxel + xentuzumab compared with control treatment group (Fig. 6e). However, only the combination treatment of paclitaxel + xentuzumab significantly reduced the average metastatic lesion size and the overall metastatic burden (Fig. 6f–h).

Taken together, our findings indicate that IGF-1 and 2 are highly expressed by both macrophages and fibroblasts in invasive breast cancer, and that blockade of IGF potentiates the efficacy of paclitaxel (Fig. 6i).

## Discussion

Here we show that IGF-1 and 2 are secreted by macrophages and fibroblasts, both at the primary site and metastatic site in invasive breast cancer, and that blocking IGF increases the efficacy of paclitaxel, a chemotherapeutic agent commonly used for the treatment of invasive breast cancer (Fig. 6i). Breast cancer, and in particular TNBC, remains a highly metastatic and potentially lethal disease with a need to identify additional specific molecular targets and to develop more effective therapies [4, 5]. Although IGF signaling has been shown to support progression of HR+ and HER2+ breast cancer and the development of resistance to established therapies, its precise role in TNBC remains elusive [32]. In our TNBC model, we found that TAMs and CAFs secrete IGF-1 and 2 at both the primary site and the pulmonary metastatic site. Zhang et al. [39], previously reported that CAF-derived IGF-1 primes breast cancer cells for bone metastasis. These studies both suggest that stromal-derived IGF plays an important role in the metastatic process of breast cancer.

In this study, we find that the Insulin/IGF1R signaling pathway is activated in 78 of 90 (~ 87%) of patients with invasive breast cancer and in 45 of 51 (~ 88.2%) TNBC patients, suggesting IGF may be a promising therapeutic target for this highly aggressive breast cancer subtype. We also observed that activation of Insulin/IGF1 receptor signaling positively correlates with increased levels of macrophage infiltration and advanced tumor stage in patients, suggesting that Insulin/IGF1 receptor activation and/or stroma expression of IGF could be predictive biomarker candidates for further evaluation.

To investigate the therapeutic potential of blocking IGF signaling in invasive breast cancer, we tested the IGF-1/2 blocking antibody xentuzumab (Boehringer Ingelheim) in two pre-clinical mouse models of invasive TNBC breast cancer, which metastasize to the lungs. In both models, we find that combining xentuzumab with paclitaxel results in reduced incidence of metastasis, as well as a significant reduction of tumor cell proliferation and metastatic burden compared with monotherapy. In agreement with our findings, Gooch et al. [40], previously showed that IGF-1 promotes proliferation of paclitaxel treated cells in vitro. Interestingly, xentuzumab + paclitaxel combination treatment significantly decreases the size of metastatic lesions but not the number of foci suggesting that the combination treatment affects metastatic outgrowth rather than the metastatic seeding.

IGF1R inhibitors have been assessed in clinical trials for metastatic HR+ as well as TNBC but have shown limited success [41–46]. Two promising IGF ligand blocking antibodies, xentuzumab and MEDI-573, are currently being evaluated in clinical trials in HR+ metastatic breast cancer patients in combination with everolimus and exemestane (NCT02123823) and in hormone sensitive metastatic breast cancer in combination with letrozole (NCT01446159), respectively. In contrast to IGF1R antibodies, IGF blocking antibodies neutralize both ligands IGF-1 and IGF-2 and thereby inhibit proliferative signaling through both Insulin and IGF1 receptors without affecting insulin metabolic signaling [38, 47].

Breast cancer cells survive poorly in isolation and participate in a complex relationship with surrounding stromal and immune cells in the tumor microenvironment, which can support tumor cell survival, proliferation, and spreading to other organs [17, 20, 21, 36, 39]. CAFs and TAMs are the most abundant stromal cells in solid cancers, including breast cancer. However, different populations of CAFs and TAMs with both pro- and anti-tumorigenic functions co-exist in tumors [48–50]. Therefore, therapies aiming to specifically inhibit the tumor supporting functions of stromal cells, without affecting their anti-tumorigenic functions, may be more effective than ablation therapies in restraining tumor progression [26, 51]. Our findings indicate that blocking IGFs in combination with paclitaxel, decreases tumor cell proliferation and breast cancer pulmonary metastasis without affecting macrophage infiltration. In conclusion, this study suggests that stroma-derived IGFs support breast cancer metastasis and modulate its response to paclitaxel, providing the rationale for further evaluation of IGF blocking antibodies in combination with paclitaxel in the treatment of invasive breast cancer.

## Materials and methods

### Generation of primary PyMT-derived breast cancer cells

Py230 cells (hormone-receptor negative and HER2 low) were generated in Ellies lab (University of California San Diego, USA) and obtained from spontaneously arising tumors in MMTV-PyMT C57Bl/6 female mice by serial trypsinization and limiting dilution [52]. The mouse model used for obtaining these tumors has been described in detail previously [53, 54].

### Generation of 4T1 derived breast cancer cells

4T1 cells were purchased from the ATCC and were originally obtained from a spontaneously arising mammary tumor in BALB/cfC3H mice [55, 56]. The orthotopic model using these cells has been described in detail previously [57].

### Cell lines and culture conditions

Murine Py230 cells were cultured in DMEM/F-12 culture media supplemented with 10% FBS and supplemented with MITO serum extender (Corning #355006), 1% penicillin/streptomycin at 37 °C, in a 5% CO_2_ incubator. Murine 4T1 cells were cultured in RPMI-1640 culture media supplemented with 10% FBS, 1% penicillin/streptomycin at 37 °C, in a 5% CO_2_ incubator. Human MDA-MB231 breast cancer cells were cultured in DMEM supplemented with 10% FBS, 1% penicillin/streptomycin, at 37 °C, 5% CO_2_ incubator. Cells were authenticated, and periodically tested for mycoplasma contamination.

### Generation of primary macrophages

Primary human macrophages were obtained from blood samples of healthy volunteers. Magnetic bead affinity chromatography was performed to purify CD14+ monocytes as per manufacturer’s instructions (Miltenyi Biotec, Woking, UK). Monocytes were incubated in RPMI media with 10% FBS and 50 ng/mL recombinant human M-CSF (Peprotech, London, UK) for 5 days post purification.

### Syngeneic orthotopic breast cancer models

Two orthotopic syngeneic breast cancer models were used in these studies. In the first breast cancer model (Fig. 5), 2 × 10^6^ Py230 luciferase/zsGreen labeled cells were injected into the fatpad of the third mammary gland of C57BL/6 6–8 week-old female mice. In the second model (Fig. 6), 5 × 10^5^ 4T1 luciferase/zsGreen labelled cells were injected into the fatpad of the third mammary gland of BALB/c 6–8 week-old female mice. Tumors were measured with calipers twice a week and treatment was started when tumors started to grow and measured between 5 and 8 mm mean diameter. Mice were administered i.p with IgG isotype control antibody, paclitaxel (100 mg/kg), IGF-1/2 blocking antibody xentuzumab (100 mg/kg) [38] kindly provided by Boehringer Ingelheim, or Paclitaxel with xentuzumab, twice a week for 15 days. At humane endpoint, primary tumors and lungs were harvested, imaged using IVIS technology and tissues were either digested for FACS sorting and analysis (see details below) or formalin-fixed and paraffin-embedded. Paraffin-embedded lungs were serially sectioned through the entire lung. Sections were stained with H&E, and images were taken using a Zeiss Observer Z1 Microscope. Number of foci and total area of metastatic foci were calculated to estimate seeding and metastasis burden using ZEN imaging software.

### FACS sorting and analysis of tumors

Tumor cells, TAMs, and stromal cells from murine primary breast tumors and pulmonary metastasis were analyzed and sorted using flow cytometry (FACS ARIA II, BD Bioscience, CA, USA). Single cell suspensions were prepared as previously described [22]. Cells were stained with Sytox blue viability marker (Life Technologies, Warrington, UK) and conjugated antibodies against anti-CD45-PE/Cy7 (Biolegend, Cambridge, UK, clone 30-F11) and anti-F4/80-APC (Biolegend, clone BM8) and analyzed using FACS Canto II (BD Biosciences).

### Gene expression

Total RNA was isolated from FACS sorted tumor cells, TAMs and stromal cells from primary breast tumors and lung metastasis. RNA extraction and cDNA were performed as previously described [22]. Gene-specific Qiagen QuantiTect Assay primers were used for qPCR analysis and relative expression levels were normalized to *gapdh* expression using the formula < 2^− (Ct *gene of interest* − Ct *gapdh*) [58].

### Gene expression analysis in TCGA database

We analyzed the Cancer Genome Atlas (TCGA) data for association between gene expression levels of *Igf1, Igf2, cd163*, and *mrc1* and 5-year overall survival. The R/Bioconductor package ‘TCGA2STAT’ (v1.2) [59] was used to download the Illumina HiSeq RNAseqV2 mRNA expression and clinical data for 1097 Breast cancer samples from the TCGA data portal. The clinical data set was filtered down to contain only those breast cancer cases classified as “infiltrating/invasive ductal carcinoma”, resulting in a reduced data set of 879 patients. For each of the candidate genes, we assessed two groups of patients; one consisting of those expressing the gene in the top 10% of expression and another containing patients expressing the gene in the bottom 10%. We compared their survival using a log-rank test at 5% significance, using the ‘survival’ (v2.41-3) package and plotted Kaplan–Meier curves for our ‘high’ and ‘low’ gene expression groups using ‘survminer’ (v0.4.0) within the ‘TCGAbrowser’ (v0.1.0) toolkit, reporting the *p*-value from the log-rank test (see Fig. 2c).

### Tissue microarrays

A TMA containing 75 breast cancer samples from consented patients was purchased from the Liverpool Tissue Bank. This TMA did not provide information of cancer subtypes. A second TMA BR10011 containing 90 invasive breast cancer samples was purchased from US Biomax. Among these 90 invasive breast cancer samples 51 were TNBC, 13 were HR+, and 19 were HER2+. Both TMAs were subjected to immunohistochemical staining and scoring by a pathologist. Detailed information of both TMAs is provided in Supplementary Tables S1 and S2.

### Immunohistochemistry and immunofluorescence

An automated DAKO PT-link (DAKO, Ely, UK) was used for deparaffinization and antigen retrieval of paraffin-embedded human and mouse breast tumors and lung metastasis. Tissues were immuno-stained using the DAKO envision + system-HRP.

#### Antibodies and procedure used for immunohistochemistry

All primary antibodies were incubated for 2 h at room temperature diluted in DAKO envision kit antibody diluent: Mouse CD68 (Abcam, Cambridge, UK, ab31630 used at 1:100 after low pH retrieval). Human CD68 (DAKO, clone KP1, M081401-2), CD163 (Abcam, ab74604), Phospho-Insulin/IGF-1R (R&D, Abingdon, UK, AF2507), phospho-insulin receptor (Lifespan Biosciences, Nottingham, UK, LS-C177981), phospho-IGF-1R (Biorbyt, Cambridge, UK, orb97626), IGF-1 (Abcam, ab9572), IGF-2 (Abcam, ab9574), CD206 (Abcam, ab8918), dilutions and conditions used as previously described [22]. Subsequently, samples were incubated with secondary HRP-conjugated antibody (from DAKO envision kit) for 1 h at room temperature. Staining was developed using diamino-benzidine and counterstained with hematoxylin.

#### Antibodies and procedure used for Immunofluorescence on paraffin-embedded tissues

Tissue sections were incubated overnight at RT with the following primary antibodies F4/80 (Biolegend, #123102 used at 1:200 after low pH antigen retrieval), Ki67 (Abcam ab15580 used at 1:1000 after low pH antigen retrieval) αSMA (Abcam ab7817 used at 1:100, after low pH antigen retrieval). Samples were washed with PBS and incubated with goat anti-rat 488 (Abcam ab96887), goat anti-rabbit 594 (Abcam ab98473) and goat anti mouse 488 (Abcam ab98637) secondary antibodies respectively all used at 1:300 and DAPI at 1:600 for 2 h at RT. Slides were washed with PBS, final quick wash with distilled water and mounted using DAKO fluorescent mounting media.

### Statistical methods

Statistical analysis for in vitro assays and animal studies was performed using unpaired two-tailed Student's *t*-test or one-way ANOVA coupled with Bonferroni’s post hoc tests, and the GraphPad Prism 5 program. All error bars indicate s.d. of *n* = 3 (in vitro studies) or SEM *n* = 3–9 (animal studies). Human samples were analyzed using Fisher’s exact test and the Matlab version 2006b program. For animal studies the group size was calculated by power analysis using a significance level kept at 5% and the power at 80% (according to approved corresponding Home Office Project License Application).

### Institutional approvals

Human tissue studies adhered to national guidelines and were approved by the University of Liverpool. Human breast cancer tissues were either patient-consented excess material obtained from the Liverpool Tissue Bank or purchased from US Biomax. Mice were maintained under specific pathogen-free conditions and experiments were performed under an approved project licence (reference number: 403725) as according to current UK legislation, at the Biomedical Science Unit at the University of Liverpool. Blood collection studies were approved by the National Research Ethics (Research Integrity and Governance Ethics committee- Reference: RETH000807). Informed consent for blood donation was given from each volunteer based on approved institutional protocols.

We thank Dr. Ilaria Malanchi for reading the manuscript and providing critical feedback. We also thank Professor Azzam Taktak for assistance with biostatistical analysis. We thank Dr. Arthur Taylor and Professor Patricia Murray for transducing the Py230 and 4T1 cells with zsGreen/luciferase lentivirus and Valeria Quaranta and Carolyn Rainer for assisting with FACS sorting. We thank Dr. Isabelle Tancioni for her technical advice with the 4T1 model. We acknowledge the Liverpool Tissue Bank for providing tissue samples, the flow cytometry/cell sorting facility, the biomedical science unit and the pre-clinical in vivo imaging facility for provision of equipment and technical assistance. We thank the patients and their families, as well as the healthy blood donors who contributed with tissue samples and blood donations to these studies.

## Electronic supplementary material

Supplementary Table 1

Supplementary Table 2

Supplementary Figure legends

Supplementary Figure 1

Supplementary Figure 2

Supplementary Figure 3

Supplementary Figure 4
