# Supplementary material for: Blockade of insulin-like growth factors increases efficacy of paclitaxel in metastatic breast cancer
Source: Oncogene. 2018 Jan 25;37(15):2022–36. doi: 10.1038/s41388-017-0115-x (PMC5895608; doi:10.1038/s41388-017-0115-x)
Supplement: Supplementary file 1 — Supplementary Table 1 [file 41388_2017_115_MOESM1_ESM.pdf]

Number	T stage	N stage	M stage	Tumor stage
1	1c	1bi	x	2A
2	2	0	x	2A
3	2	1bii	x	2B
4	1c	1bi	x	2A
5	2	0	x	2A
6	2	0	x	2A
7	2	1bi	x	2B
8	2	0	x	2B
9	1c	1bi	x	2A
10	3	1bi	x	3A
11	2	0	x	2A
12	1c	0	x	1
13	1c	1bi	x	2A
14	1c	0	x	1
15	1b	0	x	1
16	1c	1bi	x	2A
17	2	1bi	x	2B
18	1c	x	x	Not staged
19	2	1bii	x	2B
20	1c	0	x	1
21	2	1bi	x	2B
22	3	1bii	x	3A
23	1c	X	x	Not staged
24	1c	0	x	1
25	2	1bi	x	2B
26	2	0	x	2A
27	1c	0	x	1
28	1c	0	x	1
29	2	0	x	2A
30				Not staged
31	4	1bi	x	3B
32	3	1bi	x	3A
33	1c	0	x	1
34	3	1bi	x	3A
35	2	0	x	2A
36	2	0	x	2A
37	2	1bii	x	2B
38	2	1bi	x	2B
39	1c	0	x	1
40	1c	0	x	1
41	3	1bii	X	3A
42	2	1bi	x	2B
43	2	1bii	x	2B
44	1c	0	x	1
45	1c	0	x	1
46	1c	0	x	1
47	2	0	x	2A
48	1b	0	x	1
49				Not staged
50	Recurrent cancer therefore not staged			Not staged
51	1c	1bii	x	2A
52	2	1bi	x	2B
53	2	0	x	2A
54	1c	0	x	1
55	2	1a	x	2B
56	2	0	x	2A
57	1c	1bii	x	2A
58	2	x	x	Not staged
59	1c	0	x	1
60	2	1bii	x	2B
61	2	1bii	x	2B
62	2	1bi	x	2B
63	3	1bii	x	3A
64	2	0	x	2A
65			x	Not staged
66	3	1bii	x	3A
67	2	1bii	x	2B
68	2	0	x	2A
69	1c	0	x	1
70	3	0	x	2B
71	2	0	x	2A
72	2	0	x	2A
73	1c	0	x	1
74	1c	0	x	1
75				Not staged

pInsulin/IGFR - / CD163 -

pInsulin/IGFR - / CD163 +

pInsulin/IGFR + / CD163 -

pInsulin/IGFR + / CD163 +

Table S1. Clinical information from breast cancer tissue samples.

Table describing the clinical information from the 75 breast cancer consented patient samples obtained from the Liverpool Tissue bank and analysed by immunohistochemistry for phospho-Insulin/IGF1 receptor expression on cancer cells and macrophage infiltration.
