# Supplementary material for: Blockade of insulin-like growth factors increases efficacy of paclitaxel in metastatic breast cancer
Source: Oncogene. 2018 Jan 25;37(15):2022–36. doi: 10.1038/s41388-017-0115-x (PMC5895608; doi:10.1038/s41388-017-0115-x)
Supplement: Supplementary file 2 — Supplementary Table 2 [file 41388_2017_115_MOESM2_ESM.pdf]

pInsulin/IGFR + / CD163 -

pInsulin/IGFR + / CD163 +

pInsulin/IGFR - / CD163 -

pInsulin/IGFR - / CD163 +

Non-scorable scores

position	sex	age	organ	pathology	grade	stage	tnm	er	pr	Her2
A1	F	57	Breast	Invasive ductal carcinoma	1	IIA	T2N0M0	-	-	+
A2	F	39	Breast	Invasive ductal carcinoma	1	IIA	T2N0M0	-	-	+++
A3	F	28	Breast	Invasive ductal carcinoma	1	IIB	T3N0M0	+	-	-
A4	F	44	Breast	Invasive ductal carcinoma (sparse)	1	IIA	T2N0M0	-	-	-
A5	F	45	Breast	Invasive ductal carcinoma	1	IIA	T2N0M0	-	-	-
A6	F	35	Breast	Invasive ductal carcinoma	1	IIA	T2N0M0	-	-	++
A7	F	72	Breast	Invasive ductal carcinoma	2	IIA	T2N0M0	*	*	*
A8	F	50	Breast	Invasive ductal carcinoma	2	IIA	T2N0M0	-	-	-
A9	F	40	Breast	Invasive ductal carcinoma	2	IIA	T2N0M0	-	-	-
A10	F	49	Breast	Invasive ductal carcinoma	1	IIA	T2N0M0	-	-	++
B1	F	51	Breast	Invasive ductal carcinoma	2	IIA	T2N0M0	-	+	-
B2	F	44	Breast	Invasive ductal carcinoma with necrosis	3	IIA	T2N0M0	-	-	-
B3	F	65	Breast	Invasive ductal carcinoma	2	IIB	T2N1M0	-	-	-
B4	F	53	Breast	Invasive ductal carcinoma	2	IIB	T3N0M0	-	-	-
B5	F	79	Breast	Invasive ductal carcinoma with necrosis (sparse)	-	IIB	T3N0M0	-	-	+++
B6	F	50	Breast	Invasive ductal carcinoma (sparse)	-	IIB	T3N0M0	-	-	+++
B7	F	53	Breast	Invasive ductal carcinoma	2	IIB	T3N0M0	*	*	*
B8	F	30	Breast	Invasive ductal carcinoma	2	IIB	T3N0M0	-	-	++
B9	F	38	Breast	Invasive ductal carcinoma	-	IIB	T3N0M0	-	-	-
B10	F	42	Breast	Invasive ductal carcinoma	2	IIB	T3N0M0	-	-	++
C1	F	50	Breast	Invasive ductal carcinoma	2	IIB	T3N0M0	-	-	-
C2	F	50	Breast	Invasive ductal carcinoma	2	IIB	T2N1M0	-	-	-
C3	F	48	Breast	Invasive ductal carcinoma	2	IIB	T3N0M0	-	-	-
C4	F	60	Breast	Invasive ductal carcinoma	2	IIB	T3N0M0	-	-	-
C5	F	54	Breast	Invasive ductal carcinoma	2	IIB	T3N0M0	+++	-	-
C6	F	47	Breast	Invasive ductal carcinoma	2	IIB	T3N0M0	-	-	-
C7	F	41	Breast	Invasive ductal carcinoma	2	IIB	T3N0M0	-	-	+
C8	F	58	Breast	Invasive ductal carcinoma	2	IIB	T3N0M0	-	-	+++
C9	F	46	Breast	Invasive ductal carcinoma with necrosis	2	IIB	T3N0M0	-	-	-
C10	F	49	Breast	Invasive ductal carcinoma with necrosis	3	IIB	T3N0M0	-	-	-
D1	F	55	Breast	Invasive ductal carcinoma	2	IIB	T3N0M0	-	-	+++
D2	F	59	Breast	Invasive ductal carcinoma	2	IIA	T2N2M0	+	-	+++
D3	F	40	Breast	Invasive ductal carcinoma	2	IIA	T2N2M0	-	-	+++
D4	F	64	Breast	Invasive ductal carcinoma	2	IIA	T3N1M0	-	-	+++
D5	F	57	Breast	Invasive ductal carcinoma	2	IIA	T3N1M0	-	-	-
D6	F	52	Breast	Invasive ductal carcinoma with necrosis (sparse)	2	IIA	T3N1M0	*	*	*
D7	F	46	Breast	Invasive ductal carcinoma	2	IIA	T3N2M0	-	-	-
D8	F	47	Breast	Invasive ductal carcinoma	2	IIA	T3N2M0	*	*	*
D9	F	47	Breast	Invasive ductal carcinoma	2	IIB	T4N0M0	-	-	++
D10	F	85	Breast	Invasive ductal carcinoma	2	IIB	T4N0M0	-	-	-
E1	F	69	Breast	Invasive ductal carcinoma	2	IIB	T4N0M0	+++	-	-
E2	F	61	Breast	Invasive ductal carcinoma	2	IIB	T4N0M0	-	-	-
E3	F	62	Breast	Invasive ductal carcinoma	2	IIB	T4N0M0	-	-	++
E4	F	81	Breast	Invasive ductal carcinoma	2	IIB	T4N0M0	+++	-	-
E5	F	79	Breast	Invasive ductal carcinoma	2	IIB	T4N0M0	-	-	-
E6	F	40	Breast	Invasive ductal carcinoma	2	IIB	T4N0M0	-	-	-
E7	F	60	Breast	Invasive ductal carcinoma	2	IIB	T4N1M0	-	-	-
E8	F	79	Breast	Invasive ductal carcinoma	2	IIB	T4N1M0	-	-	-
E9	F	52	Breast	Invasive ductal carcinoma	2	IIB	T4N1M0	-	-	-
E10	F	71	Breast	Invasive ductal carcinoma	2	IIB	T4N1M0	-	-	-
F1	F	45	Breast	Invasive ductal carcinoma	2	IIB	T4N1M0	-	-	++
F2	F	37	Breast	Invasive ductal carcinoma	2	IIB	T4N1M0	-	-	++
F3	F	73	Breast	Invasive ductal carcinoma	2	IIB	T4N1M0	+	-	-
F4	F	41	Breast	Invasive ductal carcinoma	2	IIB	T4N1M0	-	-	-
F5	F	45	Breast	Invasive ductal carcinoma	2	IIB	T4N1M0	++	-	-
F6	F	42	Breast	Invasive ductal carcinoma	3	IIB	T4N2M0	-	-	-
F7	F	80	Breast	Invasive ductal carcinoma	2	IIA	T3N1M0	-	-	-
F8	F	50	Breast	Invasive ductal carcinoma	2	IIA	T4N1M0	-	-	++
F9	F	29	Breast	Invasive ductal carcinoma	2	IIA	T2N0M0	-	-	-
F10	F	53	Breast	Invasive ductal carcinoma with necrosis	3	IIA	T2N0M0	-	-	-
G1	F	27	Breast	Invasive ductal carcinoma with necrosis	3	IIA	T2N0M0	-	-	-
G2	F	46	Breast	Invasive ductal carcinoma	3	IIA	T2N0M0	-	-	-
G3	F	50	Breast	Invasive ductal carcinoma	3	IIA	T2N0M0	+	-	-
G4	F	43	Breast	Invasive ductal carcinoma	3	IIA	T2N0M0	-	-	-
G5	F	38	Breast	Invasive ductal carcinoma	3	IIA	T2N0M0	-	-	-
G6	F	66	Breast	Invasive ductal carcinoma with necrosis	3	IIA	T2N0M0	-	-	-
G7	F	63	Breast	Invasive ductal carcinoma	3	IIA	T2N0M0	-	-	-
G8	F	33	Breast	Invasive ductal carcinoma	3	IIA	T2N0M0	-	-	-
G9	F	53	Breast	Invasive ductal carcinoma	3	IIA	T2N0M0	-	-	-
G10	F	39	Breast	Invasive ductal carcinoma	2	IIB	T2N1M0	-	-	-
H1	F	42	Breast	Invasive ductal carcinoma	-	IIB	T2N1M0	*	*	*
H2	F	52	Breast	Invasive ductal carcinoma	-	IIB	T3N0M0	-	-	-
H3	F	41	Breast	Invasive ductal carcinoma	-	IIB	T3N0M0	*	*	*
H4	F	54	Breast	Invasive ductal carcinoma	3	IIB	T3N0M0	+++	-	+
H5	F	46	Breast	Invasive ductal carcinoma	3	IIB	T3N0M0	-	-	-
H6	F	86	Breast	Invasive ductal carcinoma	3	IIB	T3N0M0	-	-	-
H7	F	42	Breast	Invasive ductal carcinoma	3	IIB	T3N0M0	-	-	-
H8	F	50	Breast	Invasive ductal carcinoma	3	IIB	T3N0M0	-	-	-
H9	F	44	Breast	Invasive ductal carcinoma	3	IIA	T2N2M0	-	-	-
H10	F	31	Breast	Invasive ductal carcinoma	2	IIA	T3N1M0	+	-	-
I1	F	48	Breast	Invasive ductal carcinoma	3	IIB	T4N0M0	-	-	-
I2	F	49	Breast	Invasive ductal carcinoma	-	IIB	T4N0M0	*	*	*
I3	F	46	Breast	Invasive ductal carcinoma	-	IIB	T4N0M0	*	*	*
I4	F	46	Breast	Invasive ductal carcinoma	3	IIB	T4N2M0	-	-	-
I5	F	52	Breast	Invasive ductal carcinoma	2	IIB	T4N0M0	-	-	-
I6	F	47	Breast	Invasive ductal carcinoma	2	IIB	T4N0M0	-	-	-
I7	F	78	Breast	Invasive ductal carcinoma	3	IIB	T4N1M0	-	-	-
I8	F	52	Breast	Invasive ductal carcinoma	2	IIB	T4N1M0	-	-	++
I9	F	60	Breast	Invasive ductal carcinoma	2	IIB	T4N2M0	-	-	++
I10	F	70	Breast	Invasive ductal carcinoma	2	IIB	T4N2M0	++	-	-
J1	F	48	Breast	Invasive ductal carcinoma	3	IIB	T4N2M0	++	+	-
J2	F	33	Breast	Invasive ductal carcinoma	3	IIC	T3N3M0	-	-	-
J3	F	54	Breast	Invasive ductal carcinoma	3	IIC	T3N3M0	-	-	-
J4	F	41	Breast	Invasive ductal carcinoma	-	IIB	T4N0M0	-	-	-
J5	F	47	Breast	Invasive ductal carcinoma	-	IIB	T4N0M0	*	+	-
J6	F	48	Breast	Invasive ductal carcinoma	-	IIA	T2N0M0	-	-	-
J7	F	55	Breast	Invasive ductal carcinoma	-	IIA	T2N0M0	-	-	-
J8	F	64	Breast	Invasive ductal carcinoma	-	IIA	T2N0M0	-	-	-
J9	F	48	Breast	Invasive ductal carcinoma	3	IIA	T3N1M0	*	*	-
J10	F	37	Breast	Invasive ductal carcinoma	2	IIB	T3N0M0	-	-	-

Table S2: Table describing the clinical information of a tissue microarray (BR10011, U.S Biomax) containing samples from patients with invasive breast cancer from different subtypes. Tissue samples were analysed by immunohistochemistry for phospho-Insulin/IGF1 receptor expression on cancer cells and for macrophage infiltration.
