# Supplementary material for: Blockade of insulin-like growth factors increases efficacy of paclitaxel in metastatic breast cancer
Source: Oncogene. 2018 Jan 25;37(15):2022–36. doi: 10.1038/s41388-017-0115-x (PMC5895608; doi:10.1038/s41388-017-0115-x)
Supplement: Supplementary file 3 — Supplementary Figure legends [file 41388_2017_115_MOESM3_ESM.pdf]

Figure S1: Gating strategy used to FACS-sort tumor cells, tumor associated macrophages and stromal cells.

(A) Gating strategy used to sort CD45-/zsGreen+ Py230 breast cancer cells, CD45-/zsGreen- non immune stromal cells, CD45+F4/80+ macrophages from mouse breast tumors. **(B)** Quantification of αSMA mRNA expression levels in the different cell populations isolated from breast tumors using flow cytometry (n=3).

Figure S2: Macrophages are a major source of IGF in breast cancer

(A) Immunohistochemical staining of IGF-1 and IGF-2 in biopsies from patients with invasive breast cancer, scale bar 50 µm. **(B)** Quantification of Igf-1 mRNA expression levels in MDA-MB-231 human breast cancer cells and in primary human macrophages. Error bars represent s.d. (n=4), *** two tailed p value ≤ 0.005 using a student's t- test. **(C)** Quantification of Igf-2 mRNA expression levels in MDA-MB-231 human breast cancer cells and in primary human macrophages. Error bars represent s.d. (n=4), *** two tailed p value ≤ 0.005 using a student's t- test.

Figure S3: Tumor growth, tumor cell death and number of tumor infiltrated macrophages are not significantly altered with any treatment.

(A) Graph showing tumor mean diameter (mm2) measured by calipers before and during treatment with IgG control, xentuzumab, paclitaxel and paclitaxel with xentuzumab. **(B)** Quantification of dead tumor cells in tumors treated with IgG control, xentuzumab, paclitaxel and paclitaxel with xentuzumab. **(C)** Quantification of

F4/80+ macrophages among CD45+ cells in tumors treated with IgG control, xentuzumab, paclitaxel and paclitaxel with xentuzumab.

Figure S4: Insulin and IGF-1 receptor activation is decreased with xentuzumab treatment

(A) Immunohistochemical staining of phospho-insulin/IGF-1R in 4T1 breast tumors treated with human IgG (control), paclitaxel, xentuzumab or paclitaxel + xentuzumab. Scale bar 100 μm.
