# Supplementary figures and images for: Blockade of insulin-like growth factors increases efficacy of paclitaxel in metastatic breast cancer
Source: Oncogene. 2018 Jan 25;37(15):2022–36. doi: 10.1038/s41388-017-0115-x (PMC5895608; doi:10.1038/s41388-017-0115-x)

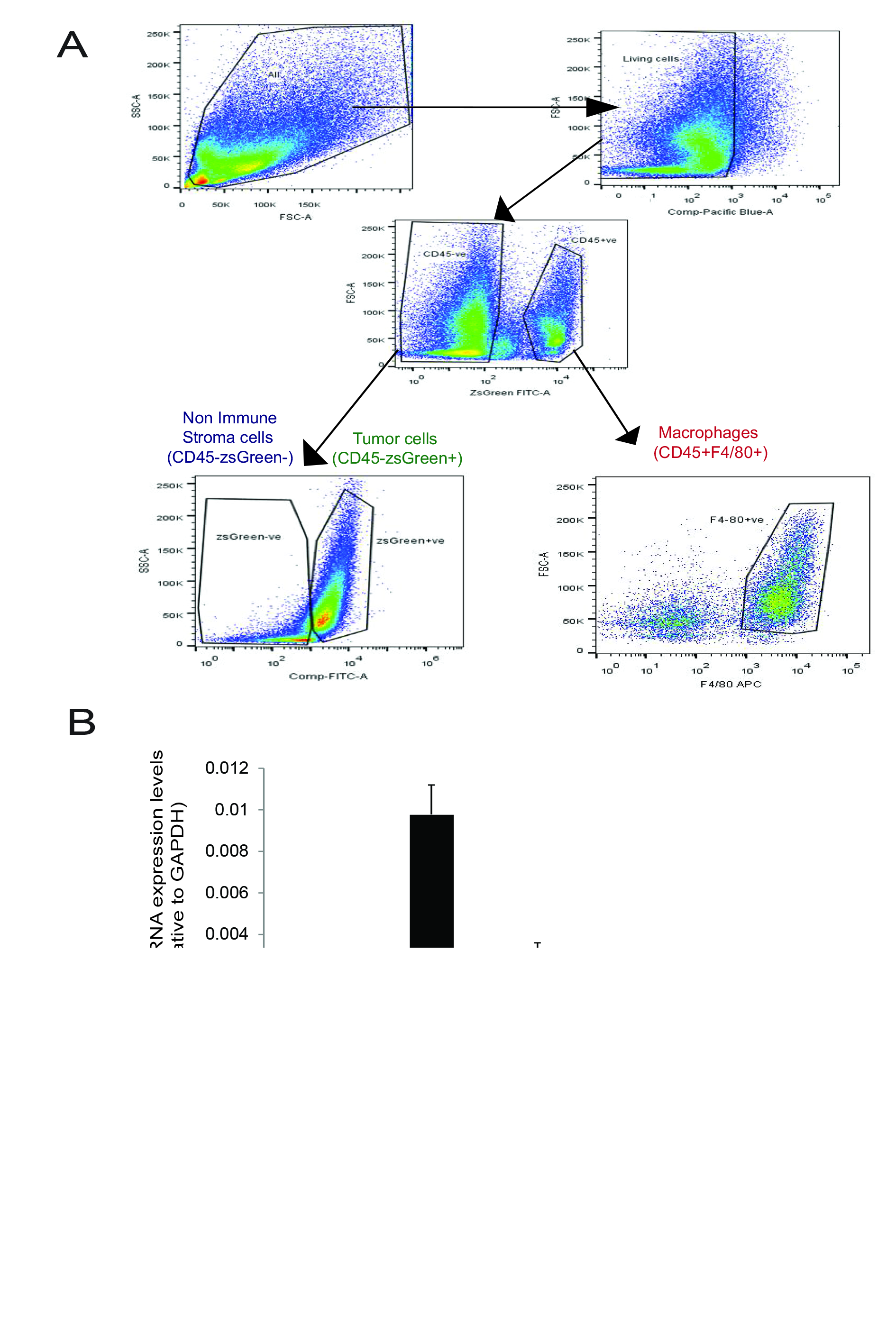

Supplement: Supplementary file 4 — Supplementary Figure 1 [file 41388_2017_115_MOESM4_ESM.tif]

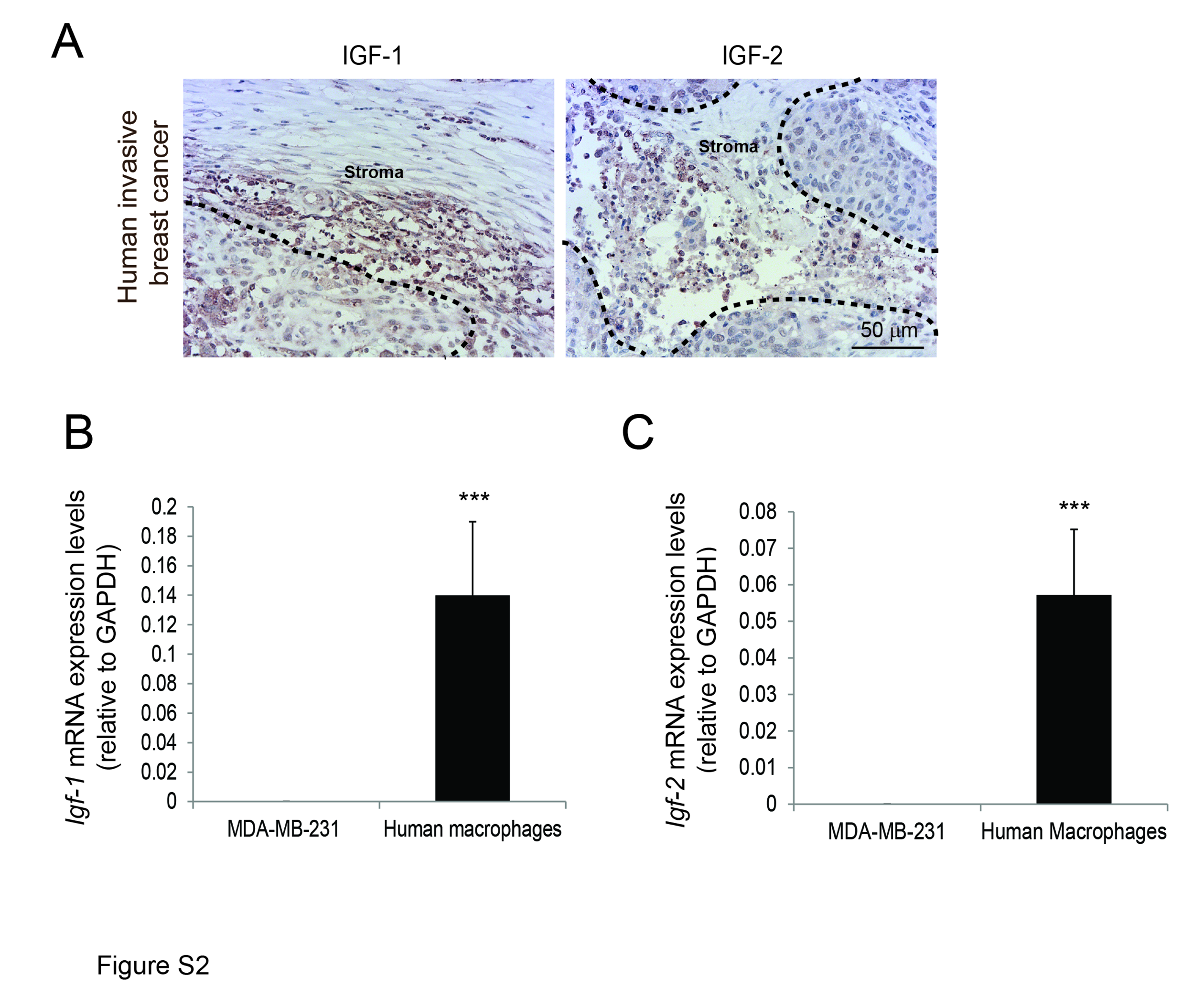

Supplement: Supplementary file 5 — Supplementary Figure 2 [file 41388_2017_115_MOESM5_ESM.tif]

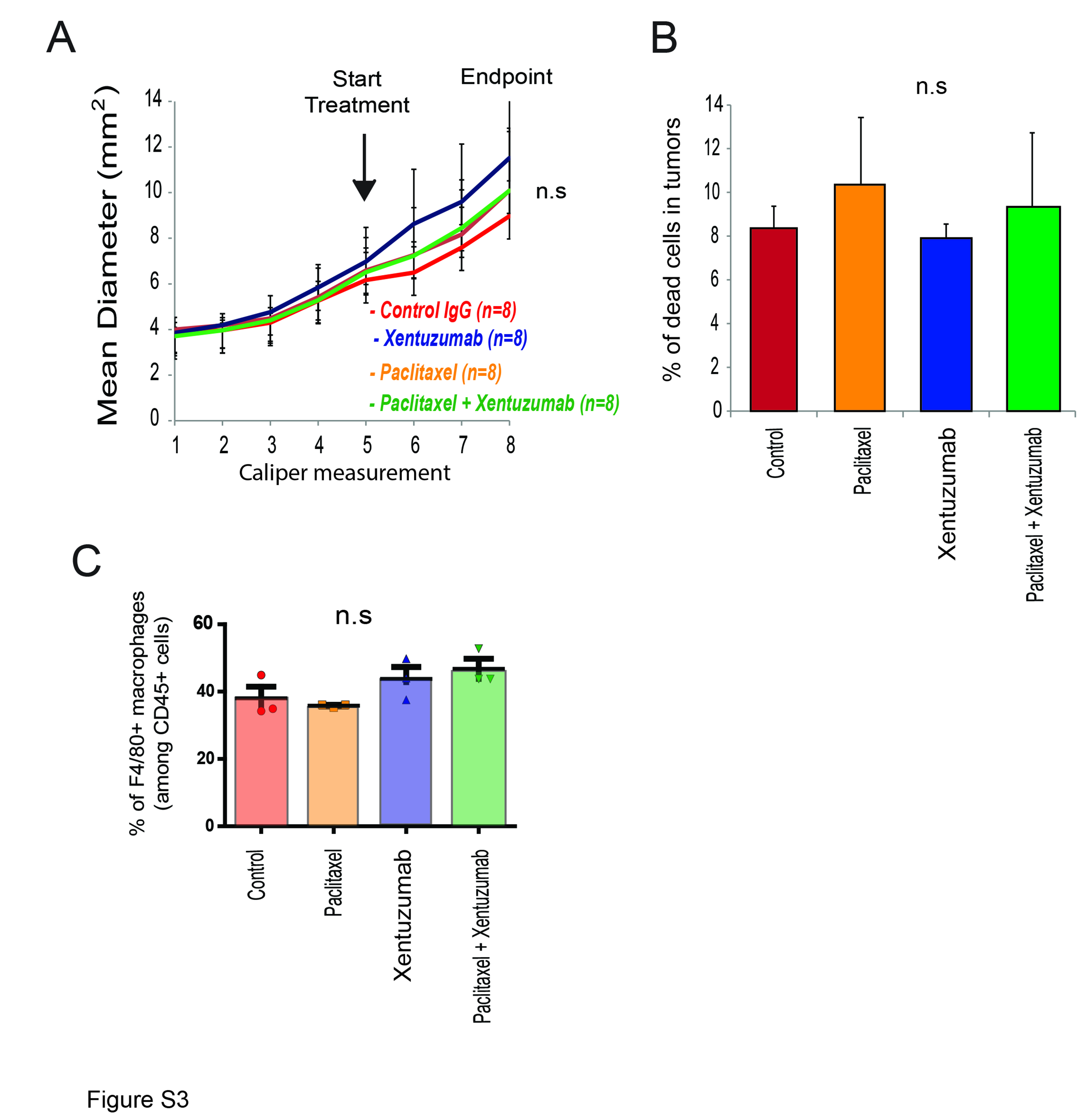

Supplement: Supplementary file 6 — Supplementary Figure 3 [file 41388_2017_115_MOESM6_ESM.tif]

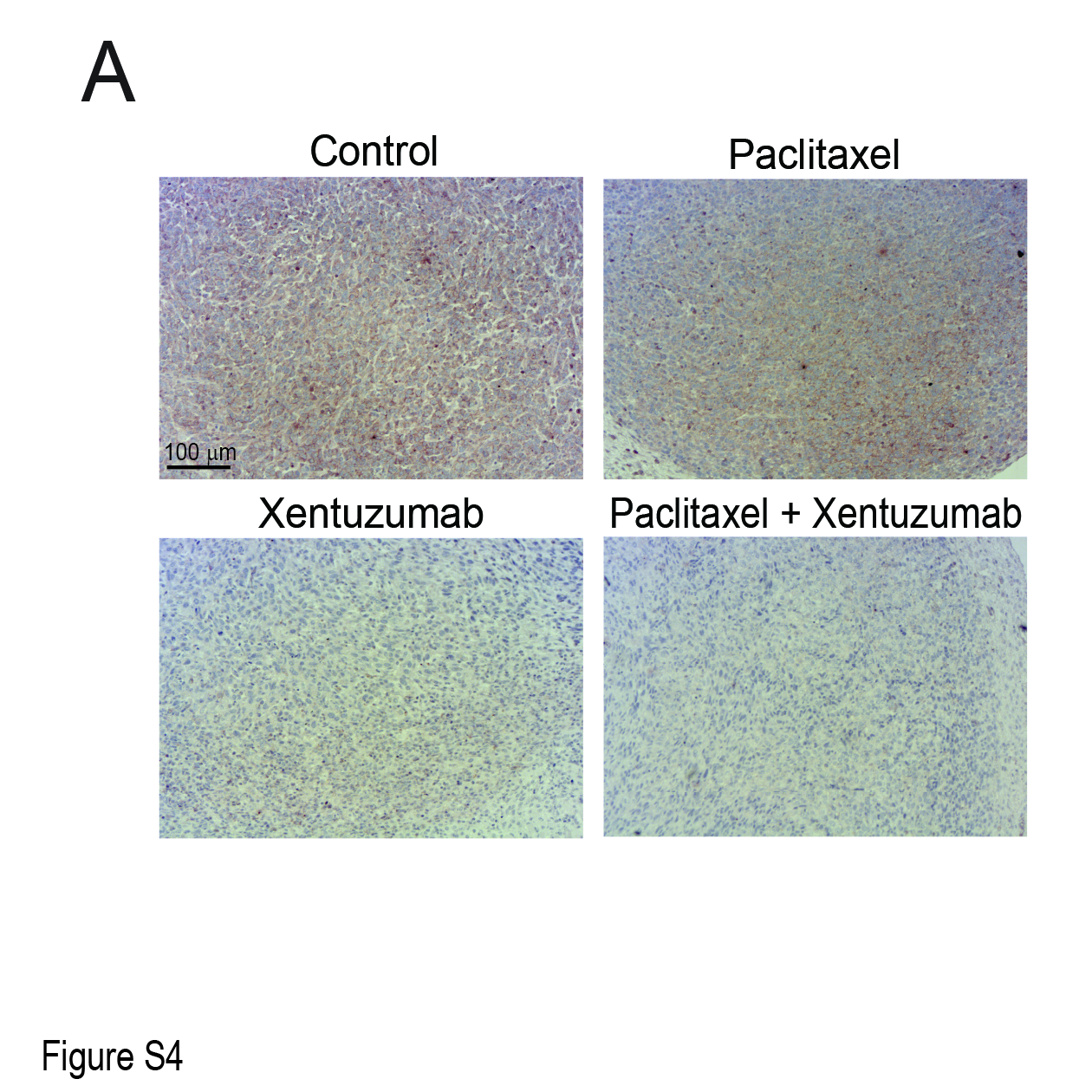

Supplement: Supplementary file 7 — Supplementary Figure 4 [file 41388_2017_115_MOESM7_ESM.tif]
